# Supplementary material for: Improved draft reference genome for the Glassy-winged Sharpshooter (Homalodisca vitripennis), a vector for Pierce’s disease
Source: G3 (Bethesda). 2021 Jul 20;11(10):jkab255. doi: 10.1093/g3journal/jkab255 (PMC8496328; doi:10.1093/g3journal/jkab255)
Supplement: jkab255_Supplementary_Data [file jkab255_supplementary_data.docx]

**Supplementary Material**

Improved draft reference genome for the Glassy-winged Sharpshooter (*Homalodisca vitripennis*), a vector for Pierce's disease

Cassandra L. Ettinger^*,1^, Frank J. Byrne^†^, Matthew A. Collin^‡§^, Derreck Carter-House^*^, Linda L. Walling^‡§^, Peter W. Atkinson^†§^ , Rick A. Redak^†^, Jason E. Stajich^*,§,2^

^*^ Department of Microbiology and Plant Pathology, University of California, Riverside, Riverside, CA, United States

^†^ Department of Entomology, University of California, Riverside, Riverside, CA, United States

^‡^Department of Botany and Plant Sciences, University of California, Riverside, CA, United States

^§^ Institute for Integrative Genome Biology, University of California, Riverside, Riverside, CA, United States

**Corresponding authors:**

^1^ Cassandra L Ettinger, Department of Microbiology and Plant Pathology, 900 University Ave, Riverside, CA 92521 [cassande@ucr.edu](mailto:cassande@ucr.edu)

^2^ Jason E Stajich, Department of Microbiology and Plant Pathology, 900 University Ave, Riverside, CA 92521 [jason.stajich@ucr.edu](mailto:jason.stajich@ucr.edu)

**Supplemental Tables:**

**Table S1. Repeat content summary.** Repeat content results are reported here including the percentage of the genome identified and masked per type repeat element by RepeatMasker. Abbreviations of repeat element categories: long-interspersed nuclear element (LINE), small-interspersed nuclear element (SINE), long-terminal repeat retrotransposon (LTR), DNA transposons (DNA), and rolling-circle transposons (RC).

| **Type of repeat element** |  | **Percent of genome** |
| --- | --- | --- |
| **SINES** |  | 0.06 |
|  | ALUs | 0 |
|  | MIRs | 0 |
| **LINES** |  | 6.67 |
|  | LINE1 | 0 |
|  | LINE2 | 3.87 |
|  | L3/CR1 | 0.51 |
| **LTR elements** |  | 0.95 |
|  | ERVL | 0 |
|  | ERVL-MaLRs | 0 |
|  | ERV-classI | 0.01 |
|  | ERV_classII | 0.03 |
| **DNA elements** |  | 5.84 |
|  | hAT-Charlie | 0.1 |
|  | TcMar-Tigger | 0.1 |
| **Unclassified** |  | 18.03 |
| **Total interspersed repeats** |  | 31.55 |
| **Small RNA** |  | 0.36 |
| **Satellites** |  | 0.01 |
| **Simple repeats** |  | 1.07 |
| **Low complexity** |  | 0.17 |

| **Gene name** | **Category** | **Flybase ID** | **Alternative species ortholog ID** | **Alternative species** |
| --- | --- | --- | --- | --- |
| **white** | Eye color marker | FBgn0003996 | [CLEC000648](https://vectorbase.org/vectorbase/app/record/gene/CLEC000648) | *Cimex lectularius* |
| **brown** | Eye color marker | FBgn0000241 | [ACYPI008444](http://bipaa.genouest.org/apps/grs-2.3/grs?reportID=aphidbase_transcript_report&objectID=ACYPI008444) | *Acyrthosiphon pisum* |
| **scarlet** | Eye color marker | FBgn0003515 | [CLEC004040](https://vectorbase.org/vectorbase/app/record/gene/CLEC004040) | *Cimex lectularius* |
| **punch** | Eye color marker | FBgn0003162 | [CLEC005231](https://vectorbase.org/vectorbase/app/record/gene/CLEC005231) | *Cimex lectularius* |
| **purple** | Eye color marker | FBgn0003141 | [CLEC001054](https://vectorbase.org/vectorbase/app/record/gene/CLEC001054) | *Cimex lectularius* |
| **sepia** | Eye color marker | FBgn0086348 | [CLEC008904](https://vectorbase.org/vectorbase/app/record/gene/CLEC008904) | *Cimex lectularius* |
| **cinnabar** | Eye color marker | FBgn0000337 | [CLEC025106](https://vectorbase.org/vectorbase/app/record/gene/CLEC025106) | *Cimex lectularius* |
| **rosy** | Eye color marker | FBgn0003308 | [CLEC006546](https://vectorbase.org/vectorbase/app/record/gene/CLEC006546) | *Cimex lectularius* |
| **vermillion** | Eye color marker | FBgn0003965 | [CLEC006165](https://vectorbase.org/vectorbase/app/record/gene/CLEC006165) | *Cimex lectularius* |
| **ebony** | Body color marker | FBgn0000527 | [CLEC007608](https://vectorbase.org/vectorbase/app/record/gene/CLEC007608) | *Cimex lectularius* |
| **curly** | Wing shape marker | FBgn0283531 | [CLEC009522](https://vectorbase.org/vectorbase/app/record/gene/CLEC009522) | *Cimex lectularius* |
| **miniature** | Wing shape marker | FBgn0002577 | [CLEC002209](https://vectorbase.org/vectorbase/app/record/gene/CLEC002209) | *Cimex lectularius* |
| **vestigal** | Wing shape marker | FBgn0003975 | [ACYPI34460](http://bipaa.genouest.org/apps/grs-2.3/grs?reportID=aphidbase_transcript_report&objectID=ACYPI34460) | *Acyrthosiphon pisum* |
| **bar** | Eye shape marker | FBgn0011758 | [CLEC007883](https://vectorbase.org/vectorbase/app/record/gene/CLEC007883) | *Cimex lectularius* |
| **PolyUbiquitin A** | Promoter of interest | FBpp0073035 | Bta02851 | *Bemisia tabaci* |
| **Actin 1** | Promoter of interest | NA | Bta13437 | *Bemisia tabaci* |
| **Actin 2** | Promoter of interest | NA | Bta08780 | *Bemisia tabaci* |
| **Actin 4** | Promoter of interest | NA | Bta00995 | *Bemisia tabaci* |
| **Exuperantia (exu)** | Promoter of interest | FBgn0000615 | [ACYPI005096](http://bipaa.genouest.org/apps/grs-2.3/grs?reportID=aphidbase_transcript_report&objectID=ACYPI005096) | *Acyrthosiphon pisum* |
| **Vasa ATP-dependent RNA helicase** | Promoter of interest | FBgn0283442 | NA | NA |
| **β-Tubulin at 60D** | Promoter of interest | FBgn0003888 | [ACYPI001007](http://bipaa.genouest.org/apps/grs-2.3/grs?reportID=aphidbase_transcript_report&objectID=ACYPI001007#.) | *Acyrthosiphon pisum* |
| **β-Tubulin at 56D** | Promoter of interest | FBgn0284243 | [ACYPI008874](http://bipaa.genouest.org/apps/grs-2.3/grs?reportID=aphidbase_transcript_report&objectID=ACYPI008874#.) | *Acyrthosiphon pisum* |
| **β-Tubulin at 85D** | Promoter of interest | FBgn0003889 | [AAEL002851](https://vectorbase.org/vectorbase/app/record/gene/AAEL002851) | *Aedes aegypti* |
| **β-Tubulin at 97EF** | Promoter of interest | FBgn0003890 | [CLEC007800](https://vectorbase.org/vectorbase/app/record/gene/CLEC007800) | *Cimex lectularius* |
| **Tub2B** | Promoter of interest | FBgn0052396 | NA | NA |

**Table S2. Reference alleles used to identify candidate genes.** For each candidate gene, we provide the gene name, the FlyBase ID (*Drosophila melanogaster,* <https://flybase.org/>) for the gene used, an alternative insect species ortholog ID used and the species the alternate ID represents. We also provide the category of interest for each gene. Broadly, these fall into two larger groupings: (1) promoter of interest or (2) a morphological marker category based on phenotype from the literature (e.g. eye color, body color, wing shape, eye shape).
